# Seroprevalence of *Toxoplasma gondii* Among Asymptomatic Children Attending Bugando Medical Centre in Mwanza, Tanzania

**DOI:** 10.1155/ijpe/3549120

**Published:** 2026-07-24

**Authors:** Delfina R. Msanga, Sofia Ottaru, Patrick Ngoya, Tulla Masoza, Rogatus Kabyemera, Domenica Morona, Stephen E. Mshana, Mariam M. Mirambo

**Affiliations:** ^1^ Department of Pediatrics and Child Health, Catholic University of Health and Allied Sciences, Mwanza, Tanzania, bugando.ac.tz; ^2^ Department of Radiology, Catholic University of Health and Allied Sciences, Mwanza, Tanzania, bugando.ac.tz; ^3^ Department of Medical Parasitology and Entomology, Catholic University of Health and Allied Sciences, Mwanza, Tanzania, bugando.ac.tz; ^4^ Department of Microbiology and Immunology, Catholic University of Health and Allied Sciences, Mwanza, Tanzania, bugando.ac.tz

**Keywords:** IgG, IgM, Mwanza, seroprevalence, *Toxoplasma gondii*

## Abstract

**Background:**

Toxoplasmosis infection affects at least 30% of the human population globally. Transmission primarily occurs through the ingestion of *Toxoplasma gondii* oocysts. Various studies have documented the seroprevalence of *T. gondii* among children with neurological manifestations such as autism, epilepsy, and hydrocephalus; however, data on asymptomatic infants remain limited. Therefore, this study is aimed at determining the seroprevalence of *T. gondii* infection and identify associated risk factors among children in Mwanza, Tanzania.

**Methods:**

This was a hospital‐based cross‐sectional study conducted between September 2017 and April 2018 in Mwanza, Tanzania. A structured and pretested questionnaire was used to capture sociodemographic, clinical data, and environmental exposure factors for *T. gondii* infection for the enrolled infants and the mother or caregivers. Clinical data were retrieved from the electronic hospital information management system. *T. gondii* specific IgG and IgM antibodies were detected by indirect enzyme‐linked immunosorbent assay (ELISA). Logistic regression analyses were used to test for associations.

**Results:**

A total of 223 infants were enrolled, with a median age (IQR) of 3 (1–7) months; 55.6% were male. Mothers or caretakers had a median age (IQR) of 27 (22–32) years. The majority were aged < 35 years, resided in urban areas, and were married. Most infants were exclusively breastfed. Good hygiene practices, including handwashing and appropriate latrine waste disposal, were commonly reported among mothers or caretakers. None of the infants tested positive for *T. gondii*–specific IgM antibodies, whereas 83 (37.2%; 95% CI: 30.0%–43.0%) were seropositive for IgG antibodies. On multivariate logistic regression analyses, having a peasant mother or caretaker (OR = 0.3, 95% CI: 0.1–0.8, *p* = 0.02) decreases the odds of being *T. gondii* IgG seropositive.

**Conclusion:**

More than one‐third of asymptomatic infants in Mwanza were seropositive for *T. gondii* IgG antibodies. The odds of *T. gondii* IgG seropositivity were significantly lower among infants whose mothers or caretakers were peasants.

## 1. Introduction

Toxoplasmosis is a global health concern affecting at least 30% of the human population worldwide [1]. Transmission primarily occurs via ingestion of *Toxoplasma gondii* oocysts often via feline contact or contaminated environments, or through consumption of bradyzoites in undercooked meat [2]. The resulting *T. gondii* infection is typically subclinical or may cause mild influenza‐like symptoms in immunocompetent hosts [3]. Severe clinical presentations may occur in the immunocompromised [2, 4].

The risk of vertical transmission during pregnancy also increases significantly with trimester [5, 6]. Furthermore, clinical symptoms of congenital infection may manifest with estimated risks rising from 25% to 65% by the third trimester [7].If left untreated during the neonatal period, affected infants are at high risk of developing long‐term neurological sequelae, including seizures and cognitive, visual, and auditory impairments [8]. Rapid diagnosis and treatment of congenital toxoplasmosis significantly reduces the risk of neurological sequelae [9, 10].

Local studies highlight a significant *T. gondii* burden, with IgG seroprevalence rates of 31%, 55.4%, and 68.4% among pregnant women, spontaneous abortion cases, and HIV individuals, respectively [11–13]. Despite available literature on the risk factors for toxoplasmosis, interestingly, some literature shows that most infected women (61%) were not exposed to the typical risk factors for infection such as exposure to raw meat or cat litter [14, 15]. Consequently, in high‐prevalence zones, such as Tanzania, establishing routine serological screening for pregnant women is vital. The seroprevalence of *T. gondii* in children with neurological manifestations such as autism, epilepsy, and hydrocephalus has been published [16–18] but limited studies have reported on asymptomatic children. Therefore, this study is aimed at evaluating the seroprevalence of *T. gondii* antibodies and identify associated risk factors in asymptomatic children in Mwanza, Tanzania.

## 2. Methodology

### 2.1. Study Design, Duration, and Setting

This was a hospital cross‐sectional study conducted between September 2017 and April 2018 at Bugando Medical Centre (BMC) in Mwanza, Tanzania. BMC is a referral and teaching hospital serving about 16 million people [19].

### 2.2. Study Population and Selection Criteria

Asymptomatic infants attended the pediatric clinic with an age of ≤ 1 year and were included. Infants that were symptomatic or with known neurological abnormalities were excluded.

### 2.3. Sample Size and Sampling Technique

A minimum required sample size of 125 infants was calculated assuming an IgG seropositivity of 8% and an IgM seropositivity of 0.115% at a 95% confidence level [18].

### 2.4. Data Collection and Procedures

Sociodemographic, clinical, and environmental exposure data related to *T. gondii* infection was collected using a structured questionnaire. The electronic Hospital Information Management System (eHIMS) was used to retrieve clinical data. As per the standard operating procedures of an accredited BMC laboratory, 3 mL of venous blood was collected from each participant using plain vacutainer tubes (Neomedic Limited, China). A calibrated and validated indirect enzyme‐linked immunosorbent assay (ELISA) (PishtazTeb Diagnostics, Iran) was used to detect *T. gondii* specific IgM and IgG antibodies. The assay had a sensitivity and specificity of 100% and 99%, respectively, for IgM detection, and 100% sensitivity and specificity for IgG detection [20]. A cutoff index value of > 1.1 was considered positive for both IgM and IgG antibodies.

### 2.5. Data Management and Analysis

Data were entered into Microsoft Excel (Microsoft, United States) and subsequently exported to STATA Version 17 (StataCorp LLC, United States) for cleaning and analysis. Continuous variables were summarized using mean and standard deviation (SD) or median and interquartile ranges (IQR), as per distribution. Categorical variables presented as frequencies or percentages. Logistic regression analysis was used to assess associations between variables. Factors with a *p* value < 0.05 in univariate analysis were included in the multivariate model to adjust for potential confounders. A *p* value < 0.05 was considered statistically significant.

## 3. Results

### 3.1. Sociodemographic and Clinical Characteristics of Enrolled Children

A total of 330 infants were assessed for eligibility, whereas 107 infants were excluded due to known neurological abnormalities. We enrolled 223 infants with a median age (IQR) of 3 (1–7) months, and 124 (55.6%) were male. One‐third (33.2%) of infants were malnourished, 35 (15.7%) had delayed or regressed developmental milestones, and 23 (10.3%) had fever. Mothers or caretakers had a median age (IQR) of 27 (22–32) years. Most mothers or caretakers 185 (83.0%) were less than 35 years of age, resided in urban settings 171 (76.7%), and were married 192 (86.1%) (Table 1).

**Table 1:** Sociodemographic and clinical characteristics of enrolled children (*n* = 223).

Variable	Frequency (*n*)	Percentage (%)
*Infant age group*		
0–6 months	163	73.1
7–12 months	60	26.9
*Infant gender*		
Female	99	44.4
Male	124	55.6
*Infant malnutrition status*		
Not malnourished	149	66.9
Malnourished	74	33.2
*Infant developmental milestones*		
Normal	188	84.3
Delayed or regressed	35	15.7
*Fever* (> 37.5°C)		
No	200	89.7
Yes	23	10.3
*Infant history of chronic disease*		
No	209	93.7
Yes	14	6.3
*Maternal age groups* ^a^		
< 35 years	185	83.0
≥ 35 years	38	17.0
*Maternal parity* ^a^		
Primipara	65	29.2
Multipara	158	70.9
*Marital status* ^a^		
Married	192	86.1
Single	31	13.9
*Residence* ^a^		
Urban	171	76.7
Rural	52	23.3
*Education level* ^a^		
College/university	20	9.0
Secondary school	61	27.4
Primary school	142	63.7
*Employment status* ^a^ (*n* = 219)		
Employed	100	45.1
Not employed	69	31.1
Peasant	53	23.9

^a^Mother or caregiver characteristic.

### 3.2. Environmental Exposures of Enrolled Children

The majority of infants, 152 (68.2%), were exclusively breastfed. A small proportion of infants started crawling 23 (10.4%), ate inedible food 26 (11.8%), and lived with cats 84 (37.7%). Handwashing practice 201 (90.1%) and latrine waste disposal 165 (74.0%) were prominent among the mothers and caretakers, and almost one‐third (32.9%) did not boil food (Table 2).

**Table 2:** Environmental exposures of enrolled children (*n* = 223).

Variable		Frequency (*n*)	Percentage (%)
*Infant*			
Exclusive breastfeeding	No	71	31.8
	Yes	152	68.2
Eats inedible food (*n* = 220)^a^	No	194	88.2
	Yes	26	11.8
Crawling (*n* = 221)	No	198	89.6
	Yes	23	10.4
Lives with cats	No	139	62.3
	Yes	84	37.7
Lives with other animals^b^	No	126	56.5
	Yes	97	43.5
*Mother or caretaker*			
Handwashing	No	22	9.9
	Yes	201	90.1
Latrine waste disposal	No	58	26.0
	Yes	165	74.0
Eats chicken (*n* = 221)	No	172	77.8
	Yes	49	22.2
Eats duck meat (*n* = 220)	No	216	98.2
	Yes	4	1.8
Eats beef	No	34	15.2
	Yes	189	84.8
Eats goat meat (*n* = 222)	No	203	91.4
	Yes	19	8.6
Eats pork	No	215	96.4
	Yes	8	3.6
Eats sheep meat (*n* = 220)	No	216	98.2
	Yes	4	1.8
Eats fish (*n* = 220)	No	211	95.9
	Yes	9	4.1
Eats vegetables	Cooked for long (≥ 5 min)	127	57.0
	Cooked briefly (< 5 min)	95	42.6
	Raw	1	0.5
Boils food (*n* = 219)	No	147	67.1
	Yes	72	32.9
Boils drinking water	No	101	45.3
	Yes	122	54.7
Does gardening	No	172	77.1
	Yes	51	22.9

^a^Soil, paper, and/or soap.

^b^Chicken, cows, dogs, pigs, and/or ducks.

### 3.3. Seroprevalence of *T. gondii*–Specific Antibodies

All 223 (100%) infants were seronegative *T. gondii–*specific IgM, whereas 83 (37.2%, 95% CI: 30.0%–43.0%) were found to be *T. gondii–*specific IgG seropositive. The median (IQR) IgG index was 0.83 (0.59–1.97).

### 3.4. Risk Factors Associated With *T. gondii* IgG Seropositivity

On multivariate regression analyses, having a peasant mother or caretaker (OR = 0.3, 95% CI: 0.1–0.8, *p* = 0.02) decreases the odds of being *T. gondii* IgG seropositive (Tables 3 and Table 4).

**Table 3:** Sociodemographic and clinical risk factors associated with *Toxoplasma gondii* IgG seropositivity (*n* = 220).

Variable	IgG negative *n* (%)	IgG positive *n* (%)	Univariate OR (95% CI); *p* value	Multivariate OR (95% CI); *p* value
*Infant age group*				
0–6 months	96 (60.0)	64 (40.0)	1	
7–12 months	41 (68.3)	19 (31.7)	0.7 (0.4–1.3); 0.26	
*Infant gender*				
Female	56 (58.3)	40 (41.7)	1	
Male	81 (65.3)	43 (34.7)	0.7 (0.4–1.3); 0.29	
*Infant malnutrition status*				
Not malnourished	96 (64.9)	52 (35.1)	1	
Malnourished	41 (56.9)	31 (43.1)	1.4 (0.8–2.5); 0.26	
*Infant developmental milestones*				
Normal	118 (63.4)	68 (36.6)	1	
Delayed or regressed	19 (55.9)	15 (44.1)	1.4 (0.7–2.9); 0.40	
*Fever* (> 37.5°C)				
No	120 (60.9)	77 (39.1)	1	
Yes	17 (73.9)	6 (26.1)	0.6 (0.2–1.5); 0.23	
*Infant history of chronic disease*				
No	131 (63.6)	75 (36.4)	1	
Yes	6 (42.9)	8 (57.1)	2.3 (0.8–7.0); 0.13	
*Maternal age groups* ^a^				
< 35 years	109 (59.9)	73 (40.1)	1	
≥ 35 years	28 (73.7)	10 (26.3)	0.5 (0.2 = 1.2); 0.11	
*Maternal parity* ^a^				
Primipara	35 (54.7)	29 (45.3)	1	
Multipara	102 (65.4)	54 (34.6)	0.6 (0.4–1.2); 0.13	
*Marital status* ^a^				
Married	121 (64.0)	68 (36.0)	1	
Single	16 (51.6)	15 (48.4)	1.7 (0.8–3.5); 0.19	
*Residence* ^a^				
Urban	101 (59.4)	69 (40.6)	1	
Rural	36 (72.0)	14 (28.0)	0.6 (0.3–1.1); 0.11	
*Education level* ^a^				
College/university	14 (70.0)	6 (30.0)	1	
Secondary school	32 (53.3)	28 (46.7)	2.0 (0.7–6.0); 0.20	
Primary school	91 (65.0)	49 (35.0)	1.3 (0.5–3.5); 0.66	
*Employment status* ^a^ (*n* = 219)				
Employed	57 (57.6)	42 (42.4)	1	
Not employed	40 (58.0)	29 (42.0)	1.0 (0.5–1.8); 0.10	0.8 (0.4–1.7); 0.63
Peasant	39 (76.5)	12 (23.5)	0.4 (0.2–0.9); 0.02	**0.3 (0.1–0.8); 0.02**

*Note:* The bolded values indicate that there is a statistical significant.

Abbreviations: CI, confidence intervals; OR, odds ratio.

^a^Mother or caregiver characteristic.

**Table 4:** Environmental risk factors associated with *Toxoplasma gondii* IgG seropositivity (*n* = 220).

Variable		IgG negative *n* (%)	IgG positive *n* (%)	Univariate OR (95% CI); *p* value	Multivariate OR (95% CI); *p* value
*Infant*					
Exclusive breastfeeding	No	46 (64.8)	25 (35.2)	1	
	Yes	91 (61.1)	58 (38.9)	1.2 (0.7–2.1); 0.60	
Eats inedible foods (*n* = 220)^a^	No	117 (60.3)	77 (39.7)	1	
	Yes	20 (76.9)	6 (20.1)	0.5 (1.2–1.2); 0.11	
Crawling (*n* = 221)	No	120 (60.9)	77 (39.1)	1	
	Yes	17 (73.9)	6 (26.1)	0.6 (0.2–1.5); 0.23	
Lives with cats	No	81 (58.3)	58 (41.7)	1	
	Yes	56 (69.1)	25 (30.9)	0.6 (0.4–1.1); 0.11	
Lives with other animals^b^	No	79 (63.7)	45 (32.3)	1	
	Yes	58 (60.4)	38 (39.6)	1.2 (0.7–2.0); 0.62	
*Mother or caretaker*					
Handwashing	No	11 (52.4)	10 (47.6)	1	
	Yes	126 (63.3)	73 (36.7)	0.6 (0.3–1.6); 0.32	
Latrine waste disposal	No	33 (58.9)	23 (41.1)	1	
	Yes	104 (63.4)	60 (36.6)	0.8 (0.5–1.5); 0.55	
Eats chicken (*n* = 221)	No	104 (60.5)	68 (39.5)	1	
	Yes	33 (68.8)	15 (31.3)	0.7 (0.4–1.4); 0.30	
Eats duck meat (*n* = 220)	No	133 (61.6)	83 (38.4)	1	
	Yes	4 (100.0)	0 (0.0)	0.2 (0.09–3.3); 0.25	
Eats beef	No	21 (67.7)	10 (32.3)	1	
	Yes	116 (61.4)	73 (38.6)	1.3 (0.6–3.0); 0.50	
Eats goat meat (*n* = 222)	No	125 (61.6)	78 (38.4)	1	
	Yes	12 (70.6)	5 (29.4)	0.7 (0.2–2.0); 046	
Eats pork	No	134 (63.2)	78 (36.8)	1	
	Yes	3 (37.5)	5 (62.5)	2.9 (0.7–12.3); 0.16	
Eats sheep meat (*n* = 220)	No	134 (62.0)	82 (38.0)	1	
	Yes	3 (75.0)	1 (25.0)	0.5 (0.1–5.3); 0.60	
Eats fish (*n* = 220)	No	129 (61.1)	82 (38.9)	1	
	Yes	8 (88.9)	1 (11.1)	0.2 (0.2–1.6); 0.13	
Eats vegetables	Cooked for long (≥ 5 min)	80 (64.0)	45 (36.0)	1	
	Cooked briefly (< 5 min)	57 (60.6)	37 (39.4)	1.2 (0.7–2.0); 0.61	
	Raw	0 (0.0)	1 (100.0)	5.3 (0.2–133.0); 0.31	
Boils food (*n* = 219)	No	90 (61.2)	57 (38.8)	1	
	Yes	46 (63.9)	26 (36.1)	0.9 (0.5–1.6); 0.70	
Boils drinking water	No	64 (64.0)	36 (36.0)	1	
	Yes	73 (60.8)	47 (39.8)	1.1 (0.7–2.0); 0.63	
Does gardening	No	99 (58.6)	70 (41.4)	1	
	Yes	38 (74.5)	13 (25.5)	0.5 (0.7–2.0); 0.04	1.1 (0.07–16.3); 0.10

Abbreviations: CI, confidence intervals; OR, odds ratio.

^a^Soil, paper, and/or soap.

^b^Chicken, cows, dogs, pigs, and/or ducks.

## 4. Discussion

This study documents a high seroprevalence of *T. gondii* IgG antibodies among asymptomatic children in Mwanza, an endemic region. The findings further indicate that having a mother or caretaker engaged in peasant farming was associated with lower odds of *T. gondii* seropositivity. In this study, the seroprevalence of *T. gondii*–specific IgG antibodies was 37.2%, which is higher than a previous study conducted in Iraq that reported a seroprevalence of 24% among school‐going children [21]. The increased seroprevalence observed in the current study could be partly explained by the endemicity of *T. gondii* in the study area, whereby a high IgG seroprevalence was observed among different groups [11–13]. Warm climatic conditions as seen in our settings may favor the sporulation of oocysts [22]. Despite the majority of infants being exclusively breastfed, the observed IgG seroprevalence in the current study reflects a decline over time of the previously transferred maternal antibodies [23].

In the current study, the odds of *T. gondii* seropositivity were lower among children whose mothers or caretakers were peasants compared with employed mothers or caretakers, consistent with a previous observational study [11]. *T. gondii* seronegativity has been associated with boiling as the predominant cooking method compared with other cooking practices [16]. Most of the mothers or caretakers reported boiling food as the primary cooking method. Boiling is known to reduce the risk of *T. gondii* infection compared with other cooking methods, such as roasting, which may increase the risk of transmission due to insufficient heat exposure [24, 25]. On the contrary, a number of studies have noted an increased risk of *T. gondii* seropositivity among peasants [22, 26, 27]. This warrants further investigation to determine why the epidemiology of *T. gondii* and risk factors vary in different regions.

This study has several limitations, including a relatively small sample size, recall bias in obtaining retrospective information, the inability to distinguish between acute and chronic infection, and maternal antibody transfer, as only a single IgG test was done without follow‐up IgG testing. Future studies involving infants would benefit from a larger sample size and the use of more inclusive diagnostic methods.

## 5. Conclusion and Recommendations

More than one‐third of the asymptomatic infants in Mwanza are *T. gondii–*specific IgG seropositive, indicating endemicity of this pathogen in Mwanza, with a significant decrease in odds of being IgG seropositive in infants whose mothers or caretakers were peasants. This calls for the need to emphasize control interventions, including one health approaches.

## Author Contributions

D.M., S.O., M.M.M., R.K., and S.E.M. were involved in designing the study; D.R.M., S.O., P.N., R.K., T.M., and M.M.M. were involved in data collection; D.R.M., S.O., P.N., and M.M.M. were involved in data analysis; S.O., M.M.M., R.K., and S.E.M. interpreted the data; D.R.M., S.O., P.N., and M.M.M. wrote the first draft of the manuscript. D.M. and S.E.M. did the critical review of the manuscript. D.R.M., S.O., P.N., and M.M.M. have contributed to the work equally and should be regarded as co‐first authors.

## Funding

This study was supported by the Catholic University of Health and Allied Sciences.

## Disclosure

All authors approved the last version of the manuscript.

## Ethics Statement

Ethical clearance (CREC/210/2017) for the study was obtained from the joint CUHAS/BMC research ethics committee. Prior to recruitment, the purpose of the study was explained to the participants. Participants′ parents or guardians signed informed consent forms prior to the involvement of their infants in the study. Confidentiality was ensured and findings relevant to the child′s management were shared with clinicians who were attending the infants. This is an umbrella approval that covers a previous study [16] and the current manuscript.

## Conflicts of Interest

The authors declare no conflicts of interest.

## Data Availability

The authors confirm that the data collected have been used for the current article. All data are available upon request to the Director of Research and Innovations of the Catholic University of Health and Allied Sciences.
